# Urinary Metabolite Levels of Flame Retardants in Electronic Cigarette Users: A Study Using the Data from NHANES 2013–2014

**DOI:** 10.3390/ijerph15020201

**Published:** 2018-01-25

**Authors:** Binnian Wei, Maciej L. Goniewicz, Richard J. O’Connor, Mark J. Travers, Andrew J. Hyland

**Affiliations:** Roswell Park Comprehensive Cancer Center, Department of Health Behavior, Elm and Carlton Streets, Buffalo, NY 14263, USA

**Keywords:** electronic cigarette, electronic nicotine delivery systems, ENDS, flame retardants, biomarker

## Abstract

Evaluating the safety of e-cigarettes and making informed judgement about developing potential standards require sufficient scientific evidence. Since e-cigarettes are highly engineered products containing plastic, glass and metal parts, and e-liquids are largely different matrices, many toxic compounds which are not typical hazards for the users of combustible tobacco products (e.g., cigarettes), could exist in e-liquids, and consequently, posing potential health risk to e-cigarette users. We combined the measurements of urinary metabolites of organophosphate flame retardants (OPFRs) with questionnaire data collected in the National Health and Nutrition Examination Surveys (NHANES) from 2013 to 2014, and we compared adjusted geometric means (GM) for each biomarker in e-cigarette users with levels in non-users and users of various tobacco products using multiple regression analyses to adjust for potential confounders. We found diphenyl phosphate (DPhP), bis(1,3-dichloro-2-propyl) phosphate (BDCPP), bis(2-chloroethyl) phosphate (BCEP), and dibutyl phosphate (DBUP) were detected in all e-cigarette users. The adjusted GM of BCEP, the metabolite of tris(2-chloroethyl) phosphate (TCEP), was 81% higher than nonusers (*p* = 0.0124) and significantly higher than those for both cigarette and cigar users (*p* < 0.05). The findings in this pilot study suggest that certain OPFRs may present in e-cigarettes as contaminants, and consequently, resulting in higher exposure levels in e-cigarette users compared to nonusers. As we only identified 14 e-cigarette users in the survey, the findings in this study need to be confirmed in future study at a larger scale. A better examination of the types and levels of FRs and their potential contamination sources in e-cigarettes is also needed.

## 1. Introduction

Electronic cigarettes, often referred to as e-cigarettes or electronic nicotine delivery systems (ENDS), are engineered to heat a nicotine solution (hereafter called e-liquid) to generate and deliver inhaled nicotine-containing aerosol [1]. The commonly used carrier constituents in e-liquids are propylene glycol (PG) and vegetable glycerin (VG), which are often mixed together. Without combustion of e-liquid constituents, e-cigarette aerosol contains lower levels of toxic compounds than conventional combustible tobacco products [2]. For this reason, e-cigarettes are aggressively marketed as “safer” products, and the use of e-cigarettes has actually been increasing over the last decade in the United States and around the world [3].

Evaluating the safety of e-cigarettes requires sufficient scientific evidence, including solid measurements from comprehensive laboratory tests. Considering that e-cigarettes are highly engineered products containing plastic, glass and metal parts, and e-liquids are largely different matrices, many toxic compounds, which are not typical hazards for the users of combustible tobacco products (e.g., cigarettes and cigars), could exist in e-cigarettes and/or e-liquids and pose potential health risk to e-cigarette users. One group of such harmful chemicals is flame retardants (FRs). FRs are widely used in plastics, electronics, and other household and consumer products to reduce product flammability to meet safety standards [4]. In a recently study, Chung, Zheng [5] found elevated levels of polybrominated diphenyl ethers (PBDEs), one type of FRs, in e-cigarette aerosols. The authors made the argument that the potential source may be the PBDEs leaching from e-cigarette atomizers and the associated protective casing. There is currently no additional information available in the open literature to validate and confirm these findings. More research is needed to examine the levels of FRs in e-liquids resulting from potential contamination by unknown sources during the procedures of production, transport, and storage. Revealing such information will provide scientific data so regulators can make informed judgement about developing potential standards. Standards for maximum allowable levels of FRs may be needed considering the potential toxicity of these chemicals. FRs have been found to be endocrine-disrupting chemicals (EDCs) [6] and several FRs, including tris(1,3-dichloro-2-propyl)phosphate (TDCPP) and tris(2-chloroethyl)phosphate (TCEP), are potential carcinogens, mutagens, and neurotoxicants [4].

The specific aim of this study is to evaluate whether use of e-cigarette potentially increases the body burden of FRs in e-cigarette users. To achieve this goal, we first combined self-reported questionnaire data with urinary metabolite concentrations of organophosphate FRs (OPFRs) measured in participants of the National Health and Nutrition Examination Surveys (NHANES) during the period 2013–2014. We then categorized the participants as nonusers of any tobacco products, exclusive e-cigarette users, and other users of non-cigarette tobacco products. We finally evaluated and compared the urinary metabolite concentrations of OPFRs among those groups.

## 2. Materials and Methods

Subjects included in this analysis were aged 20 and older and participated in the NHANES from 2013 to 2014. NHANES is a cross-sectional health examination survey conducted by the National Center for Health Statistics (NCHS) of the U.S. Centers for Disease Control and Prevention (CDC) [7].

We restricted our analyses to 1572 participants whose biomarker and questionnaire data were commonly available. Within this dataset, participants were classified as nonusers if they reported not using any tobacco or nicotine products (i.e., cigarette, cigar, pipe, snuff/snus, and nicotine patch) within the five days prior to their NHANES physical examination. Exclusive e-cigarette users, exclusive cigarette smokers, exclusive cigar smokers, and exclusive users of smokeless tobacco products were identified if they self-reported use of e-cigarettes, cigarettes, cigar, and smokeless tobacco products, respectively, within the five days prior to examination but never used any other tobacco products. The sample size characteristics of each category are presented in Table 1.

Nine urinary metabolites of organophosphate FRs (Table 2) were measured at CDC’s Division of Laboratory Sciences (DLS) using a method developed by Jayatilaka, Restrepo [4]. This method utilized an ultra-high performance liquid chromatography coupled with tandem mass spectrometry with limits of detection (LODs) ranging from 0.05 to 0.16 µg/L [4,9]. Urinary creatinine concentration was measured using a colorimetric method based on a Jaffé rate reaction. According to the analytical method [4,9], strict quality control (QC) and quality assurance (QA) CLIA guidelines were followed to perform analytical measurements. Laboratory blanks and QC samples (high and low concentrations) were simultaneously processed and analyzed to assure the quality of the analytical results to meet the accuracy and precision specification of the QC/QA program of the Division of Laboratory Sciences at the US CDC [10].

We performed statistical analysis using SAS (version 9.4; SAS Institute Inc., Cary, NC, USA). We first merged the data regarding biomarker concentrations and tobacco-associated questionnaire data according to NCSH recommendations [13]. We calculate descriptive statistics using the “univariate” SAS procedure. In order to compare biomarker levels among different tobacco products users and nonusers, we performed multiple regression analyses to calculate the least square geometric mean (hereafter called the adjusted GM) and 95% confidence intervals (95% CI) for each metabolite by incorporating appropriate design effects of stratification and clustering and sampling weights [14,15]. log10-transformed creatinine (log_UCre) was included to account for variation arising from urine dilutions among spot samples [16]. Small sample sizes for non-tobacco users precluded us from testing the distributions, but it is still possible to evaluate the overall exposure tendencies among different categories [17]. Here, we used log-transformed biomarker data in all the regression analysis because of the skewed distributions observed in nonusers and cigarette users as well as in other similar biomonitoring studies [14,16,18]. Other covariates evaluated in the regression model included smoking status, age, race/ethnicity, gender, poverty, education, and all possible two-way interactions. Concentrations below the LOD were substituted with the LOD divided by the square root of two. Statistical analysis was confined to measurements with a frequency of detection greater than 60% to avoid undue influence on the estimates caused by imputed values. In all cases, using Wald’s F statistics, significance was set at *p* < 0.05.

We used backward elimination with a threshold of *p* < 0.05 for retaining covariates and two-way interactions to reach the final reduced models. In addition, we evaluated potential confounding by covariates that were not significant predictors by adding each back into a model that included significant predictors only. If the addition of one of these excluded variables caused a change of ≥10% in the β coefficient for any of the significant predictors, we re-added the variable to the model. The final regression model for diphenyl phosphate (DPhP) included the following significant predictors: log_UCre (*p* < 0.001), gender (*p* < 0.001), and the interaction of race/ethnicity and education (*p* < 0.001); the final regression model for bis(1,3-dichloro-2-propyl) phosphate (BDCPP) included the following significant predictors: log_UCre (*p* < 0.001), age (*p* < 0.001), gender (*p* < 0.05), and the interaction of race/ethnicity and education (*p* < 0.001). The final regression model for bis(2-chloroethyl) phosphate (BCEP) included the following significant predictors: log_UCre (*p* < 0.001), gender (*p* < 0.05), and the interaction of race/ethnicity and education (*p* < 0.001). The final regression model for dibutyl phosphate (DBUP) included the following significant predictors: log_UCre (*p* < 0.001), age (*p* < 0.01), gender (*p* < 0.01), and the interaction of race/ethnicity and education (*p* < 0.001).

## 3. Results

From the 2013–2014 survey, we identified 1201 nonusers of tobacco, 298 exclusive cigarette smokers, 22 exclusive cigar smokers, 14 exclusive e-cigarette users, and 15 exclusive users of smokeless tobacco products (STB) (Table 1). Among all users of different tobacco products, DPhP, BDCPP, BCEP, and dibutyl phosphate (DBUP) had the highest detection rates (Table 2); the detection rates for BCPP were generally below 60%, and those for di-p-cresyl phosphate (DpCP), di-o-cresyl phosphate (DoCP), dibenzyl phosphate (DBzP), and 2,3,4,5-tetrabromobenzoic acid (TBBA) were all <15%.

Table 3 presents the selected percentiles of DPhP, BDCPP, BCPP, BCEP, and DBUP. Percentiles of DpCP, DoCP, DBzP, and TBBA were not presented because of low detection rates. The adjusted GM of BCEP for e-cigarette users was 81% higher than nonusers (*p* = 0.0124) and significantly higher than those for both cigarette and cigar users (*p* < 0.05) (Table 4). The adjusted GM of BCEP for the users of smokeless products was approximately 46% higher than nonusers, but no significant difference between them was identified (*p* = 0.35). The STB users had a significantly higher adjusted GM of DBUP than those for nonusers (*p* = 0.011), cigarette users (*p* = 0.012), and cigar users (*p* = 0.028). The adjusted GM of DBUP for e-cigarette users was approximately 18% higher than that for nonusers, but no statistical difference was identified. The adjusted GM of DPhP for cigar users was the highest among all categories but was only statistically higher than that for the users of STB (*p* < 0.05). No significant differences were identified in the adjusted GM of DCPP among all groups.

## 4. Discussion

This study is the first to assess the exposure to FRs in exclusive users of different tobacco products using urinary FR metabolite concentrations measured in NHANES. Among all four metabolites with detection rates >60%, we observed higher adjusted GM for BCEP, a metabolite of tris(2-chloroethyl) phosphate (TCEP), in the users of e-cigarettes than those in both nonusers and cigarette users, suggesting that using e-cigarettes could lead to elevated exposure to TCEP. In a similar fashion, cigar users may have a higher exposure to TPhP, while smokeless tobacco users showed higher exposure to TBUP but lower exposure to TPhP. Although our results are preliminary, they indicate a need for a better examination of the types and levels of OPFRs and their potential contamination sources in non-cigarette tobacco products.

There is currently limited information available to compare with our results obtained using biomarker measurements in NHANES during 2013–2014. Previously, Chung, Zheng, et al. [5] found elevated levels of PBDEs in e-cigarette aerosols. The authors made the argument that the potential source may be the PBDEs leaching from e-cigarette atomizers and the associated protective casing. This might be true considering the highly engineered characteristics of e-cigarettes that contain certain plastic components. Some FRs can also be used as plasticizers or lubricants [4,12,19]. For example, TPhP and TBuP are commonly used as plasticizers and lubricants to regulate pore size [4,12,19]. In particular, there are no chemical bonds between FRs and plastic materials in which they are used. Especially, predominant organic constituents (i.e., PG or VG) in e-liquids can facilitate the leaching of FRs from the plastic parts in e-cigarettes. In addition, e-liquids can be contaminated when contacting with materials where FRs are used during manufacturing, storing, and packaging processes. These speculations can be better examined in future studies conducted on a larger scale. Investigation of the types and levels of FRs in e-cigarettes and their potential contamination sources during production, transport, storage, and use of e-cigarettes might also be important. Obtaining such information is crucial for assessing the exposure to, and health risks from, FRs when using non-cigarette tobacco products.

Our findings should be interpreted in the context of several limitations. Firstly, the limited sample sizes for non-cigarette tobacco users precluded systematic demographic analysis and comprehensive exposure characterization. The limited sample only allowed us to evaluate the overall exposure tendencies among different categories. Secondly, the metabolites of FRs have been frequently detected in general population, and none of the FRs are e-cigarette-specific compounds. The measured metabolite levels of FRs reflect the integrated contributions from all potential exposure sources. In addition, one FR metabolite, i.e., DPhP, is not a specific metabolite for TPhP. DPhP is a major metabolite and a common biomarker of aryl-PFRs. DPhP itself is also used as an additive in industrial manufacturing activities [11,12]. These concerns should be considered in future studies on identification of FR exposure sources. Thirdly, although a significantly higher level of BCEP was identified in the e-cigarette users in contrast to nonusers and users of combustible tobacco products, we were unable to determine the specific sources of FR exposure. Additional studies are needed to determine the FR levels and potential sources in the processes to produce, transport, storage, and use of e-cigarettes/e-liquids. Lastly, since current data is only available for nine FR metabolites, we were unable to evaluate any metabolites of other widely used FRs, for instance, tris(2-butoxyethyl) phosphate.

## 5. Conclusions

The findings in this study on the basis of the biomarker data measured in NHANES 2013–2014 indicate that certain OPFRs may present in e-cigarettes, resulting in higher exposure levels in e-cigarette users compared to nonusers. As we only identified 14 e-cigarette users in the survey, the findings in this study need to be confirmed in future studies conducted on a larger scale. More research is also needed to examine the types and levels of FRs in e-cigarettes/e-liquids and investigate their potential contamination sources during production, transport, storage, and use of e-cigarettes.

## Figures and Tables

**Table 1:** Demographic characteristics of study participants.

	Nonuser N = 1201	Cigarette User N = 298	Cigar User N = 22	E-Cigarette User N = 14	User of Smokeless Tobacco Products N = 15
Gender, N.					
Male	534	170	18	8	15
Female	667	128	4	6	-
Age (year), N.					
20–45	511	142	12	10	6
>46	690	156	10	4	9
Race, N.					
NH White	532	139	10	8	9
NH Black	190	78	9	1	2
Mexican American	178	28	2	-	1
Others	301	53	1	5	3
Poverty Income Ratio *^a^*, N					
PIR < 1	264	120	8	4	6
1 ≤ PIR < 1.93	271	78	4	4	2
1.93 ≤ PIR < 3.71	299	66	6	1	2
PIR ≥ 3.71	367	34	4	5	5
Education, N					
<High School	194	84	5	3	3
HS/GED	247	85	6	1	5
College or AA degree	376	105	6	8	6
College graduate or above	384	24	5	2	1

Abbreviations: NH—non-Hispanic; HS/GED—high school graduate/General Educational Development or equivalent; AA degree—an associate’s degree. *^a^* poverty income ratio (PIR) is an index calculated by dividing family income by a poverty threshold specific to family size [8].

**Table 2:** Flame retardants (FRs) and their urinary metabolites, abbreviations, and detection percentages (%).

Parent FRs	Metabolite	Nonuser	Cigarette User	Cigar User	E-Cigarette User	User of Smokeless Tobacco Products
Triphenyl phosphate (TPhP), etc.	Diphenyl phosphate (DPhP) ^a^	89.3	91.0	95.5	100	87.5
Tris(1,3-dichloro-2-propyl) phosphate (TDCPP)	Bis(1,3-dichloro-2-propyl) phosphate (BDCPP)	88.7	92.9	86.3	100	94.1
Tris(1-chloro-2-propyl) phosphate (TCPP)	Bis(1-chloro-2-propyl) phosphate (BCPP)	58.7	51.1	63.6	57.1	56.3
Tris(2-chloroethyl) phosphate (TCEP)	Bis(2-chloroethyl) phosphate (BCEP)	86.9	89.6	90.9	100	87.5
Tri-p-cresyl phosphate (TpCP)	Di-p-cresyl phosphate (DpCP)	9.4	12.4	13.6	14.3	0
Tri-o-cresyl phosphate (ToCP)	Di-o-cresyl phosphate (DoCP)	0	0	0	0	0
Tributyl phosphate (TBUP)	Dibutyl phosphate (DBUP)	78.1	82.0	86.3	100	87.5
Tribenzyl phosphate (TBzP)	Dibenzyl phosphate (DBzP)	0	0	0	0	0
2-ethylhexyl-2,3,4,5-tetrabromobenzoate (TBB)	2,3,4,5-tetrabromobenzoic acid (TBBA)	4.5	5.7	13.6	0	6.3

^a^ DPhP is a major metabolite and a common biomarker of aryl-PFRs. DPhP itself is also used as additive in industrial manufacturing activities [11,12].

**Table 3:** Selected percentiles of urinary metabolites of FRs (in µg/g creatinine).

Metabolite	Percentiles	Nonuser	Cigarette User	Cigar User	E-Cigarette User	User of Smokeless Tobacco Products
DPhP	25th	0.42	0.47	0.55	0.50	0.19
	50th	0.71	0.73	0.77	0.57	0.35
	75th	1.35	1.18	1.59	1.43	0.80
	95th	4.84	3.51	9.93	3.29	1.93
	Max	113	73.7	9.93	3.31	1.93
BDCPP	25th	0.38	0.36	0.28	0.38	0.19
	50th	0.76	0.74	0.52	0.80	0.53
	75th	1.59	1.46	1.21	3.00	1.45
	95th	4.77	3.79	1.87	4.37	1.93
	Max	56.1	67.7	2.17	4.37	1.93
BCPP	25th	<LOD	<LOD	<LOD	<LOD	<LOD
	50th	0.20	0.14	0.17	0.10	0.12
	75th	0.35	0.24	0.27	0.41	0.17
	95th	1.30	0.87	2.15	0.78	0.40
	Max	18.5	2.43	2.15	0.78	2.85
BCEP	25th	0.19	0.20	0.21	0.39	0.09
	50th	0.35	0.35	0.29	0.92	0.45
	75th	0.73	0.75	0.61	1.42	2.03
	95th	2.95	3.97	3.47	2.47	2.34
	Max	31.8	60.4	3.94	2.47	2.61
DBUP	25th	0.12	0.12	0.13	0.15	0.15
	50th	0.21	0.19	0.18	0.17	0.23
	75th	0.35	0.29	0.25	0.28	0.31
	95th	0.66	0.64	0.76	0.93	1.63
	Max	7.82	4.91	0.76	0.93	1.63

**Table 4:** Adjusted geometric means of FR metabolites among different tobacco user categories and nonusers.

Flame Retardant	Urinary Metabolite *^a^*	Adjusted Geometric Means *^b^* (95% Confidence Interval), μg/L				
		Nonuser	Cigarette User	Cigar User	E-Cigarette User	User of Smokeless Products
Triphenyl phosphate (TPhP)	DPhP *^c^*	0.70 (0.65, 0.74)	0.72	1.15	0.74	0.41
			(0.61, 0.85)	(0.68, 1.96)	(0.49, 1.12)	(0.26, 0.66)
			*p* = 0.62	*p* = 0.06	*p* = 0.74	*p* = 0.032
Tris(1,3-dichloro-2-propyl) phosphate (TDCPP)	BDCPP	0.71 (0.61, 0.81)	0.65	0.50	0.71	0.52
			(0.54, 0.77)	(0.28, 0.87)	(0.29, 1.68)	(0.36, 0.78)
			*p* = 0.27	*p* = 0.25	*p* = 0.97	*p* = 0.15
Tris(2-chloroethyl) phosphate (TCEP)	BCEP	0.37 (0.33, 0.42)	0.39	0.38	0.67	0.54
			(0.31, 0.49)	(0.22, 0.65)	(0.44, 1.04)	(0.24, 1.25)
			*p* = 0.64	*p* = 0.95	*p* = 0.012	*p* = 0.33
Tributyl phosphate (TBUP)	DBUP	0.17 (0.14, 0.20)	0.17	0.16	0.20	0.30
			(0.14, 0.21)	(0.11, 0.25)	(0.11, 0.36)	(0.18, 0.48)
			*p* = 0.97	*p* = 0.86	*p* = 0.63	*p* = 0.011

*^a^* Abbreviations: DPhP—diphenyl phosphate; BDCPP—bis(1,3-dichloro-2-propyl) phosphate; BCEP—bis(2-chloroethyl) phosphate; DBUP—dibutyl phosphate. *^b^ p*-values between exclusive tobacco users and nonusers, using Wald’s F statistic, by multiple regression analyses. The final regression model for DPHP included the following significant predictors: log_UCreatinine (*p* < 0.001), gender (*p* < 0.001), and the interaction of race/ethnicity and education (*p* < 0.001); the final regression model for BDCPP included the following significant predictors: log_UCreatinine (*p* < 0.001), age (*p* < 0.001), gender (*p* < 0.05), and the interaction of race/ethnicity and education (*p* < 0.001). The final regression model for BCEP included the following significant predictors: log_UCreatinine (*p* < 0.001), gender (*p* < 0.05), and the interaction of race/ethnicity and education (*p* < 0.001). The final regression model for DBUP included the following significant predictors: log_UCreatinine (*p* < 0.001), age (*p* < 0.01), gender (*p* < 0.01), and the interaction of race/ethnicity and education (*p* < 0.001). *^c^* DPHP is a major metabolite and a common biomarker of aryl-PFRs. DPhP itself is also used as additive in industrial manufacturing activities [11,12].
